# Monocyte-Macrophage Lineages in Cancer: Biology, Plasticity, and Therapeutic Targeting

**DOI:** 10.66505/cbtt.v1i2.23

**Published:** 2026-05-12

**Authors:** Christian A. Owusu, Ramona Moles

**Affiliations:** 1. Department of Cell and Molecular Biology, University of Mississippi Medical Center, Jackson, MS 39216, USA; 2. Cancer Center and Research Institute, University of Mississippi Medical Center, Jackson, MS 39216, USA; 3. Center for Immunology and Microbial Research, University of Mississippi Medical Center, Jackson, MS 39216, USA

**Keywords:** myeloid cells, macrophages, tumor-associated macrophages, TAM, myeloid-derived suppressor cells, MDSC, myeloid reprogramming, tumor microenvironment, cancer immunotherapy, nanomedicine

## Abstract

Monocytes are highly plastic innate immune cells that serve as critical sentinels of host defense, tissue homeostasis, and inflammatory regulation. Beyond their canonical functions in phagocytosis, cytokine and chemokine production, and antigen presentation, monocytes play increasingly recognized roles in shaping the tumor ecosystem. In cancer, malignant cells and their associated stromal networks profoundly reprogram monocyte development, trafficking, and functional states, converting these cells from immune defenders into key facilitators of tumor initiation, progression, and therapeutic resistance. Upon infiltration into the tumor microenvironment (TME), circulating monocytes differentiate into heterogeneous populations of tumor-associated macrophages (TAMs) that support cancer cell survival, angiogenesis, immune suppression, and metastatic dissemination. Recent advances in single-cell transcriptomics, spatial profiling, lineage tracing, and high-dimensional cytometry have transformed our understanding of monocyte and macrophage biology, revealing previously unappreciated diversity in their ontogeny, phenotypic states, and context-dependent functions across tumor types and disease stages. These technologies have uncovered dynamic crosstalk between monocytes, TAMs, cancer cells, and other immune and stromal components, highlighting monocyte-derived populations as central regulators of the immunosuppressive TME. In this Review, we synthesize current insights into the functional heterogeneity of monocyte and macrophage subsets in cancer, emphasizing their roles in tumorigenesis, immune evasion, and therapeutic responses. We further discuss emerging therapeutic strategies aimed at targeting monocyte recruitment, differentiation, and effector functions, including macrophage reprogramming, depletion, and combinatorial approaches with immunotherapy. Collectively, these advances position monocytes and their progeny as both biomarkers and actionable targets, offering new opportunities to reshape the tumor immune landscape for durable cancer control.

## 1. Introduction

The immune system exerts a paradoxical influence on cancer, functioning both as a barrier to malignant transformation and as an active architect of tumor progression. Central to this duality is the remarkable plasticity of myeloid cells, particularly monocytes and macrophages, whose phenotypes and functions are shaped by tissue context, metabolic cues, and inflammatory signals. Traditional binary classifications of macrophages into pro-inflammatory “M1” and anti-inflammatory “M2” states have provided a useful conceptual framework but increasingly fail to capture the complexity observed in human tumors. Monocyte-macrophage lineages refer to the continuum of circulating monocytes, tissue-resident macrophages, and tumor-associated macrophages that collectively regulate immune surveillance, tissue remodeling, and tumor progression within the tumor microenvironment. Emerging evidence shows that tumor-associated myeloid cells occupy a continuum of activation states, often co-expressing inflammatory, immunosuppressive, and tissue-remodeling programs. This functional heterogeneity is further amplified by spatial constraints within the tumor microenvironment, temporal evolution during disease progression, and therapeutic pressure. As a result, macrophage behavior cannot be reliably inferred from canonical markers or static polarization models. Recognizing the inadequacy of simplified paradigms is therefore essential for understanding how monocytes and macrophages orchestrate immune evasion, therapeutic resistance, and metastatic dissemination in cancer.

**Figure 1.**
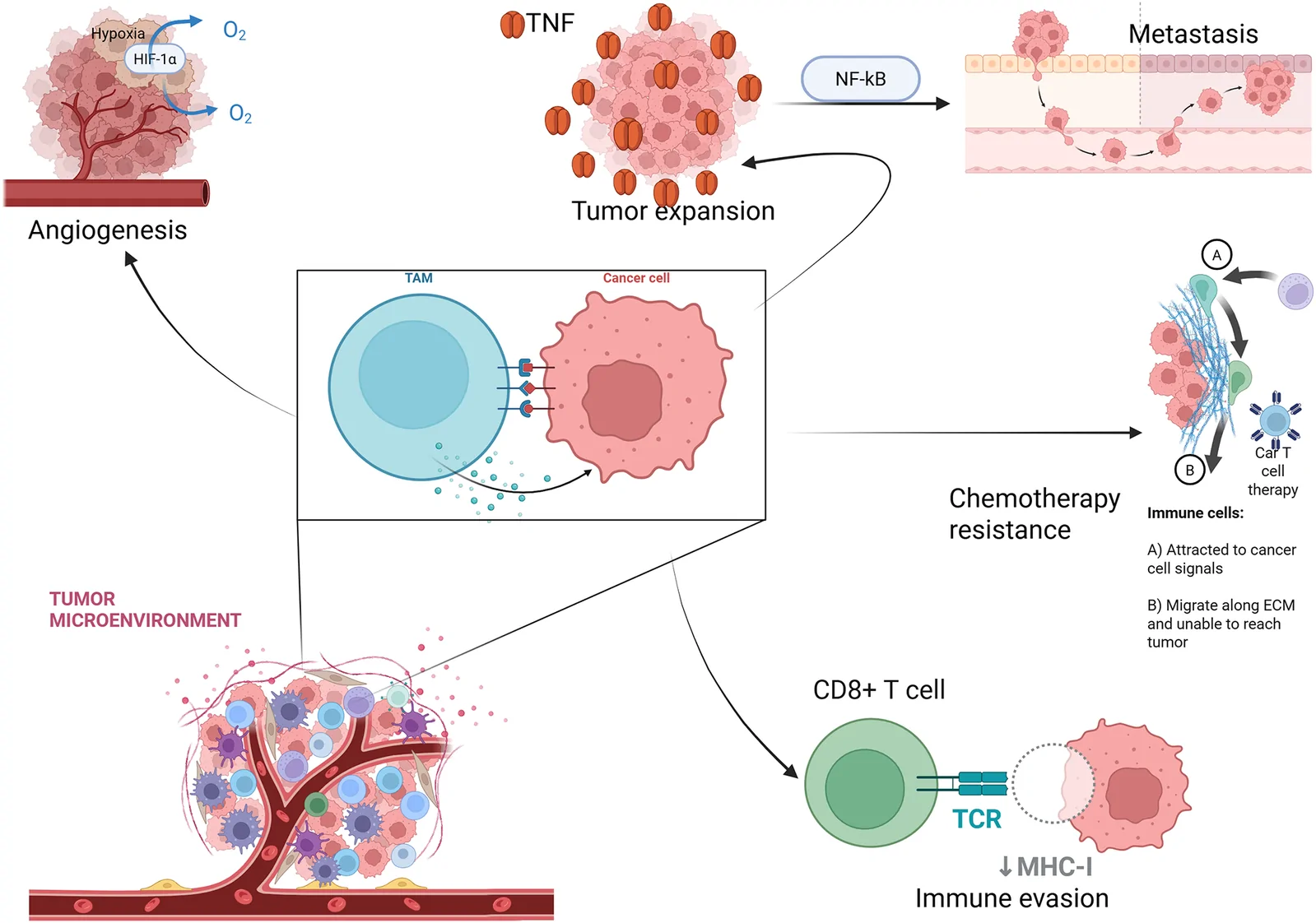
Roles of tumor-associated macrophages (TAMs) in cancer progression. Within the tumor microenvironment (TME), TAMs interact with cancer cells, promoting tumor growth and metastatic dissemination. TAM-derived tumor necrosis factor (TNF) activates nuclear factor-κB (NF-κB) signaling in cancer cells, leading to upregulation of anti-apoptotic genes and enhanced proliferation. TAMs also contribute to extracellular matrix (ECM) remodeling by depositing matrix proteins and promoting lysyl oxidase-mediated cross-linking of collagen and elastin, thereby increasing matrix stiffness that restricts infiltration by chimeric antigen receptor (CAR) T cells. In addition, TAMs secrete immunosuppressive cytokines, including interleukin-10 (IL-10) and transforming growth factor-β (TGF-β), and reduce major histocompatibility complex class I (MHC-I) expression on tumor cells, thereby impairing CD8^+^ T-cell-mediated immune surveillance. TAM-derived reactive oxygen species (ROS) promote stabilization of hypoxia-inducible factor 1α (HIF-1α), driving pro-angiogenic gene expression and neovascularization that support tumor growth and metastasis.

The interaction between tumor cells and the immune system is a central determinant of cancer development and progression. Through a complex network of cytokines, chemokines, and metabolic cues, tumors actively shape immune responses that can either restrain or promote malignant growth. Monocytes are key mediators of this crosstalk, as they are efficiently recruited to tumor sites in response to tumor- and stroma-derived signals, thereby accumulating within primary tumors and metastatic niches (1–3). These short-lived, circulating mononuclear phagocytes infiltrate tissues and differentiate into diverse myeloid populations, including myeloid-derived suppressor cells (MDSCs) and tumor-associated macrophages (TAMs), which constitute major cellular components of the tumor microenvironment (TME). Accumulating evidence indicates that these monocyte-derived populations contribute to angiogenesis, immune suppression, metastatic dissemination, and resistance to chemotherapy (4–6). While macrophages retain the capacity to eliminate tumor cells through phagocytosis, exposure to the TME frequently reprograms them toward tumor-supportive states (7, 8), underscoring their functional duality and context dependence during tumor progression (9) (Fig. 1). Together, these observations position monocyte-derived populations as central architects of the tumor microenvironment and key regulators of tumor immunity, angiogenesis, and therapeutic resistance.

Recent technological advances have fundamentally reshaped our understanding of monocyte and macrophage diversity in cancer. Single-cell transcriptomic and epigenomic profiling have revealed extensive heterogeneity within circulating monocytes and tumor-associated macrophages, uncovering discrete developmental trajectories, activation states, and functional programs that were previously obscured by bulk analyses. Spatially resolved transcriptomics and multiplex imaging further demonstrate that macrophage phenotypes are tightly coupled to their anatomical localization within the tumor microenvironment, with distinct niches supporting immunosuppressive, angiogenic, or inflammatory programs. Complementing these approaches, lineage-tracing and fate-mapping studies have clarified the ontogeny of tumor-associated macrophage populations, distinguishing monocyte-derived cells from tissue-resident macrophages and revealing context-dependent contributions to tumor growth and immune regulation. Together, these high-resolution technologies highlight that monocyte and macrophage states are dynamic, reversible, and shaped by local cues, challenging static models of myeloid identity and underscoring the need for integrated, multidimensional frameworks to interpret myeloid function in cancer.

Despite these advances, major conceptual and translational gaps remain. It remains unclear which monocyte-derived and macrophage subsets are causally linked to tumor progression, therapeutic resistance, or durable clinical responses, and how these populations evolve under treatment pressure. Furthermore, the field lacks unified criteria to functionally define myeloid subsets across tumor types and disease stages, complicating biomarker development and therapeutic targeting. Emerging strategies that modulate monocyte recruitment, reprogram macrophage states, or selectively deplete tumor-promoting subsets have shown promise, yet their clinical success has been variable, reflecting incomplete mechanistic insight and context dependence. In this Review, we synthesize current knowledge on monocyte and macrophage heterogeneity in cancer, highlight unresolved challenges in classification and functional attribution, and discuss therapeutic approaches that exploit myeloid plasticity. By integrating insights from cutting-edge technologies with clinical observations, we aim to provide a conceptual framework to guide the rational design of next-generation myeloid-targeted therapies. These advances motivate a closer examination of how innate myeloid cells are organized and reprogrammed within the tumor microenvironment. In this Review, we propose that tumor progression is strongly shaped by a dynamic myeloid plasticity network in which monocytes, macrophages, and related myeloid populations continuously adapt to tumor-derived inflammatory, metabolic, and spatial cues.

## 2. Innate Myeloid Landscapes in the Tumor Microenvironment

Innate myeloid cells constitute a dominant and functionally diverse component of the tumor microenvironment (TME), integrating tumor-intrinsic signals with local stromal and immune cues. In this section, we outline the organizational principles of innate myeloid landscapes within the TME, focusing on monocyte recruitment, macrophage reprogramming, and the microenvironmental signals that define tumor-associated myeloid states. Understanding how monocytes and macrophages are recruited, reprogrammed, and spatially organized within the tumor microenvironment provides a conceptual framework for interpreting their roles in tumor progression and therapeutic response.

Cancer progression is strongly associated with genetic and epigenetic changes, but recent studies show that TME is also a key determinant (10). TME is a niche within the body that contains both cellular and non-cellular components (11–15). Cellular components include cancer, stromal, and immune cells (such as T and B lymphocytes, neutrophils, natural killer (NK) cells, dendritic cells (DCs), MDSCs, and macrophages). Non-cellular components encompass the extracellular matrix (ECM) (collagens, fibronectin, and laminins), soluble mediators (cytokines, chemokines, and growth factors), extracellular vesicles, and physicochemical features such as hypoxia, acidity, and metabolites (10). Immune cells in the TME are not malignant but contribute to tumor suppression or progression (11, 12) through cell-surface receptor expression and production of soluble factors. Recent spatial transcriptomics analyses, often integrated with single-cell RNA sequencing, have revealed that immune and myeloid cells are organized into spatially distinct niches within the TME, shaping macrophage localization, functional states, and tumor progression (14, 15). Within the TME, monocytes are polarized into distinct phenotypes and functions (16–19). For example, Th2 signals drive macrophage differentiation toward an M2 anti-inflammatory phenotype that promotes tumor progression (20). Notably, macrophages present in the tumor site, referred to as TAMs, are transcriptionally distinct from both monocytes and their tissue-resident counterparts. This distinct identity arises from active reprogramming within the TME, where hypoxia, metabolic stress, cell-cell interactions, cytokines, and growth factors drive context-dependent differentiation programs (21). In cancer patients, altered chemokine levels, including changes in C-X3-C motif chemokine ligand 1 (CX3CL1) and reduced C-C motif chemokine ligand 2 (CCL2) in the circulation, are associated with changes in monocyte mobilization and recruitment to tumors. As a result, TAMs acquire tumor-associated transcriptional states enriched for genes involved in immune regulation, extracellular matrix remodeling, angiogenesis, and tissue repair, and are accompanied by reduced expression of genes involved in inflammatory and antigen presentation pathways (22–24).

Tumor-derived signals such as colony-stimulating factor 1 (CSF1), interleukin-10 (IL-10), transforming growth factor beta (TGF-β), hypoxia-inducible factors (HIFs), and metabolic mediators including lactate, lipids, and oxidative stress activate multiple transcriptional regulators in TAMs, including signal transducer and activator of transcription 3 (STAT3), hypoxia-inducible factor-1 alpha (HIF-1α), peroxisome proliferator-activated receptor gamma (PPARγ), and nuclear factor kappa B (NF-κB).

These pathways collectively shape TAM transcriptional programs, leading to sustained expression of genes commonly associated with tumor macrophages, such as complement component 1q (C1Q), apolipoprotein E (APOE), triggering receptor expressed on myeloid cells 2 (TREM2), galectin-3 (LGALS3), cluster of differentiation 163 (CD163), and V-set and immunoglobulin domain-containing protein 4 (VSIG4) (21). In several human cancers, TAMs also show elevated expression of sialic acid-binding immunoglobulin-like lectin 1 (SIGLEC1), which encodes cluster of differentiation 169 (CD169), and C-C motif chemokine ligand 8 (CCL8), a macrophage-derived chemokine linked to tumor progression and metastasis (25–27). In contrast, circulating monocytes maintain transcriptional programs associated with migration, pathogen sensing, and inflammatory cytokine production, whereas tissue-resident macrophages preserve lineage-specific programs related to tissue development and homeostasis (28, 29).

In this context, the TME is a complex, dynamic niche that integrates immune, stromal, and tumor-derived elements. The cellular component is primarily composed of immune cells, including T cells, TAMs, dendritic cells, NK cells, MDSCs, and neutrophils, which together define the immune compartment of the TME and are discussed in detail in later sections of this review. There are also stromal cells, such as fibroblasts and adipocytes, that can acquire altered phenotypes to promote cancer progression. Malignant tumor cells can also actively influence surrounding stromal and immune cells. The non-cellular component comprises the ECM, a network of structural proteins and bioactive molecules that forms a scaffold promoting tumor cell adhesion, migration, and invasion. In addition, bioactive molecules contribute to the chemical and physical properties of the TME. The presence of cytokines and chemokines, combined with a hypoxic environment, contributes to TME complexity and heterogeneity, thereby increasing resistance to chemotherapy (30–33). As a consequence of this organization, the TME also contributes to therapy resistance, tumor development, and metastasis, largely through immune cells and mediators such as CCL2, CXCR4, and TGF-β (34–37). This supportive niche is maintained by non-tumor cells, ECM remodeling, and soluble mediators. The ECM is frequently remodeled and stiffened, thereby serving as a physical and biochemical barrier that limits T-cell infiltration and the penetration of chemotherapeutic drugs. Nutrient-deprived conditions within the TME impose metabolic stress that favors the selection of aggressive tumor phenotypes. Cell-ECM interactions induce the release of soluble factors that promote immune evasion and ECM remodeling, thereby contributing to therapy resistance. In addition, the hypoxic nature of the niche, the presence of tumor-derived exosomes, and deregulated metabolic pathways play key roles in supporting the TME and promoting cancer progression (38–40).

Because cancer cell survival and chemotherapy resistance depend on a supportive niche, the TME has become a major focus of cancer research. Studying the microenvironment beyond tumor cells alone has revealed how interactions among cancer cells, immune cells, stromal components, and the ECM support tumor growth and therapy resistance, thereby guiding the development of therapeutic strategies that disrupt these tumor-supportive interactions and improve treatment responses in cancer patients.

### 2.1 Tumor-Driven Reprogramming of Monocyte Phenotypes

Tumor cells actively reprogram infiltrating monocytes, reshaping their phenotype, metabolic state, and differentiation potential to support immune suppression and tumor progression. This reprogramming is driven by tumor-derived cytokines, metabolic stress, and aberrant activation of intracellular signaling pathways, altering monocyte abundance, function, and clinical impact. In this section, we discuss the mechanisms by which tumors remodel monocyte phenotypes and the prognostic and therapeutic implications of these changes.

Myeloid cells are involved in homeostasis, protection against pathogens, tissue remodeling, and activation of adaptive immunity (41–43). Myeloid cell populations within the TME broadly regulate tumor elimination or survival (44). The presence of infiltrating innate myeloid immune cells, such as monocytes, DCs, and macrophages, initiates a cycle of responses at the tumor site. These cells can act as antitumor effectors or promote cancer growth (45). Tumor-associated myeloid cells include monocytes, MDSCs, tumor-associated DCs, and TAMs (46).

Monocytes, a crucial component of the innate immune system, are divided into three subpopulations in humans: classical, non-classical, and intermediate (47, 48). Originating in the bone marrow, monocytes migrate to sites of inflammation, where they differentiate into macrophages or DCs. Monocytes and their derivatives display three main functions: antigen presentation, phagocytosis, and cytokine/chemokine production (49). However, in the context of cancer, tumor cells can exploit the TME to reprogram myeloid cells to favor cancer progression. The release of tumor-associated cytokines, such as G-CSF and GM-CSF, alters the metabolic profile of myeloid cells, including upregulation of lipid transport and uptake pathways. This increased lipid uptake drives metabolic and functional reprogramming, promoting immunosuppressive mechanisms and enhancing the survival and expansion of myeloid cells within the tumor environment (50, 51). As a result, tumor-derived signals alter monocyte abundance, phenotype, differentiation trajectories, and overall immune function. Within this altered environment, monocytes are preferentially driven to differentiate into TAMs rather than into immunostimulatory dendritic cells. This skewed differentiation is mediated by tumor-derived cytokines and aberrant activation of intracellular signaling pathways. For example, ovarian cancer cells release leukemia inhibitory factor (LIF) and IL-6 into the TME, which promote TAM generation by redirecting monocyte differentiation toward TAM-like phenotypes. In parallel, tumor-induced activation of the MAPK and PI3K/AKT pathways in myeloid cells further reinforces M2-like polarization, suppressing differentiation toward antigen-presenting dendritic cells (25, 52).

Cancer development typically involves inflammation, which can be both a driver and a consequence of tumorigenesis. This inflammation recruits inflammatory cells, including monocytes, from the bloodstream to the tumor site, and tumors can also promote monocytosis by releasing cytokines and chemokines that enhance myelopoiesis and monocyte mobilization. This may contribute to increases in the total count and relative number of monocytes, which are commonly observed in cancer patients. The connection between cancer and inflammation creates a conducive environment for tumor initiation, with about 20% of cancer cases arising from infection and chronic inflammation. Chronic inflammation primarily suppresses the antitumor response and drives tissue remodeling by releasing cytokines and chemokines, such as VEGF, TGF-β, and TNF-α, and by activating pathways such as NF-κB and STAT3, thereby promoting cancer progression (53–56). Studies have shown that an elevated absolute monocyte count (AMC) at diagnosis is associated with worse overall survival. Higher circulating monocyte counts are associated with poor clinical outcomes and may reflect increased TAM activity, which is linked to angiogenesis, immunosuppression, and metastasis (Fig. 1) (53, 57–59). Ratios reflecting monocyte proportion, such as the low lymphocyte-to-monocyte ratio (LMR), predict poor outcomes in many solid tumors (53, 60–62). Pretreatment values of AMC or LMR can help identify patients at higher or lower risk. For example, elevated AMC has been linked to poor survival in prostate and pancreatic cancers (63, 64). A low LMR is associated with adverse outcomes in oral and head and neck cancers (HNCs) (65, 66). Monitoring these measures during therapy may also provide insight into treatment response; however, their clinical utility requires further validation in large, prospective studies to determine their predictive value across different tumor types and treatment settings.

Additional studies highlight the potential role of monocytes as prognostic markers. Signal regulatory protein α (SIRPα) is a receptor that suppresses monocyte/macrophage phagocytic function and inflammatory signaling. The interaction between SIRPα and cluster of differentiation 47 (CD47, the “self” signal expressed by normal cells) prevents phagocytic clearance. Consistent with this, tumor cells often overexpress CD47 to evade phagocytosis by monocytes and macrophages (67). Clinical studies further show that increased numbers of CD14^+^ SIRPα^high^ monocytes are associated with reduced survival in follicular lymphoma, whereas an increased CD14^−^ SIRPα^low^ monocyte subset correlates with better survival (68). Monocyte numbers are not only associated with cancer survival but can also predict responsiveness. Patysheva et al. reported that the percentages of CD163-expressing CD14^low^ CD16^+^ and CD14^+^ CD16^+^ monocytes are increased in breast cancer compared with healthy women; moreover, CD163^+^ monocytes increase in tumors after neoadjuvant chemotherapy (NAC), and the proportion of CD14^low^ CD16^+^ monocytes positively correlates with response to NAC (69). The TME shapes monocyte phenotypes to favor cancer progression. These alterations can serve as indicators of tumor grade and prognosis (70). In pancreatic cancer, circulating monocytes exhibit constitutive phosphorylation of signal transducer and activator of transcription (STAT) family proteins and a diminished response to stimulation, indicating abnormal activation and impaired immunity (71). Serum levels of monocyte-associated cytokines, including interleukin-6 (IL-6), granulocyte colony-stimulating factor (G-CSF), and granulocyte-macrophage colony-stimulating factor (GM-CSF), are elevated in lung cancer. Notably, increased G-CSF levels correlate with adverse clinical outcomes and are associated with advanced disease stage, increased tumor burden, and greater disease severity (72).

The direct effects of tumor cells on monocyte phenotype have been validated ex vivo. Co-culturing monocytes from cancer patients with tumor cells resulted in downregulation of the pro-inflammatory macrophage marker CD86, suggesting compromised antitumor function (71). An additional study showed increased expression of the cancer stemness-promoting factor CD51 in monocytes after co-culture with pancreatic tumor cells (73). Taken together, the TME profoundly shapes monocyte populations and phenotypes, driving their differentiation toward immunosuppressive states.

### 2.2 Monocyte-Derived DCs and MDSCs in Cancer

In addition to giving rise to tumor-associated macrophages, monocytes represent a critical source of dendritic cells and myeloid-derived suppressor cells (MDSCs) in cancer, with profound consequences for antitumor immunity and disease progression. Tumor-derived inflammatory and metabolic cues skew monocyte differentiation toward immunosuppressive MDSC and macrophage lineages rather than immunostimulatory dendritic cell lineages. In this section, we examine the mechanisms governing monocyte fate decisions in cancer and discuss how monocyte-derived DCs and MDSCs contribute to immune evasion, therapy resistance, and metastatic dissemination.

Monocytes can differentiate into DCs or macrophages in response to cytokine stimulation. DCs generally support antitumor immunity by presenting tumor-associated antigens and priming cytotoxic CD8^+^ T cells. Tumor-derived factors shift this balance. For example, retinoic acid in the TME has been reported to favor differentiation of intratumoral monocytes toward TAMs, which support tumor growth, in part by suppressing the DC-associated transcription factor IRF4 (74). Although the mechanisms remain incompletely understood, soluble factors in the TME appear to drive infiltrating monocytes toward immunosuppressive TAMs rather than toward immunostimulatory DCs. Tumor-derived cytokines and growth factors, including IL-10, IL-6, TGF-β, and vascular endothelial growth factor (VEGF), suppress DC differentiation while promoting immunosuppressive macrophage phenotype (75, 76). For example, in human melanoma, melanoma cell supernatants rich in IL-10 have been shown to induce a shift in monocyte polarization toward a CD163+, programmed death-ligand 1 (PD-L1)+ M2-like phenotype and to inhibit monocyte differentiation into DCs, which are associated with tumor progression (77). In cancer, circulating monocytes are a major source of MDSCs. Tumor-driven inflammation can reprogram these cells toward immunosuppression (41). MDSCs include granulocytic/polymorphonuclear (G-MDSCs or PMN-MDSCs) and monocytic (M-MDSCs). M-MDSCs are defined as CD11b^+^Ly6G^−^Ly6C^high^ in mice and CD11b^+^CD14^+^HLA-DR^−^/loCD15^−^ in humans, and low MHC-II further helps distinguish them from monocytes. During tumor development, persistent signals such as GM-CSF, IL-6, and IL-1β pathologically activate monocytes and drive the expansion of M-MDSCs (41, 78).

The development and suppressive function of MDSCs are regulated by multiple transcriptional and signaling pathways. Key regulators of MDSC development include cellular Rel proto-oncogene (c-Rel), STAT3, S100 calcium-binding protein A8 and S100 calcium-binding protein A9 (S100A8/9), TNF-α-induced protein 8-like 2 (TIPE2), and Interferon Regulatory Factor 8 (IRF8) (79–82). c-Rel is a central driver of M-MDSC growth and suppressive function (83). c-Rel belongs to the Nuclear Factor κ-light-chain-enhancer of activated B cells (NF-κB) family and turns on immunosuppressive enzymes and other M-MDSC genes by forming a c-Rel/ CCAAT/enhancer-binding protein β (C-EBPβ)/pSTAT3/p65 enhanceosome (79). A c-Rel-programmed subset of M-MDSCs in mouse and human melanoma, termed c-Rel-dependent monocytes (rMos), has been described. These c-Rel^+^, IL-1β^high^, Arginase-1 (Arg-1)^−^ rMos promote tumor growth by blocking T-cell function and maintaining a suppressive microenvironment through IL-1β/CCL2 crosstalk (84). MDSCs play an essential role in tumorigenesis and cancer progression by inhibiting T-cell proliferation and activation through multiple mechanisms, including cytokine modulation, metabolic depletion, and direct interactions (85, 86). For example, in acute myeloid leukemia (AML), increased M-MDSC counts are associated with poor prognosis (87). Notably, elevated M-MDSC levels predict reduced immunotherapy efficacy, including CAR-T, and are associated with chemoresistance (88, 89). Within the TME, multiple tumor-derived signals further reinforce M-MDSC suppressive phenotypes. In glioblastoma, the cell adhesion molecule integrin β1 and the dipeptidyl peptidase-4 (DPP-4), which are upregulated in M-MDSCs, act as central regulators that promote their tumor-promoting functions (90). Leukocyte immunoglobulin-like receptor B4 (LILRB4) drives the polarization of M-MDSCs and downregulates miR-1 family microRNAs, thereby promoting tumor cell migration and invasion (91, 92). Soluble Heat Shock Protein 90 α (HSP90α) can program human monocytes into immunosuppressive M-MDSCs via Toll-like Receptor 4 (TLR4) signaling, which, in turn, upregulates PD-L1 on these cells (93). Post-transcriptional mechanisms also contribute to M-MDSC function. Functional suppression of the type I interferon (IFN-I) axis is necessary for MDSCs to develop immunosuppressive activity in cancer, whereas stabilizing Interferon α/β Receptor Subunit 1 (IFNAR1) and combining it with interferon-based therapy produces a strong antitumor response (94). Finally, Ubiquitin-Specific Peptidase 12 (USP12)-mediated deubiquitination and stabilization of p65 diminish both the infiltration and suppressive capacity of M-MDSCs, enhancing CD8^+^ T-cell activity and slowing tumor growth (95).

M-MDSCs exhibit lower surface MHC-II expression than monocytes. Increased autophagy in these cells accelerates lysosomal degradation of MHC-II, thereby reducing surface expression and impairing antitumor immunity. When autophagy is reduced, lysosomal processing is diminished, MHC-II surface levels rise, and tumor-specific CD4^+^ T cells are more effectively activated. Inhibiting the E3 ligase Membrane-Associated RING-CH-type Finger 1 (MARCH 1) stabilizes MHC-II at the plasma membrane, indirectly weakening the suppressive program of M-MDSCs and leading to potent antitumor responses and marked tumor reduction (96, 97). Together, these findings indicate that the TME reprograms monocyte differentiation into M-MDSCs through coordinated transcriptional and post-transcriptional mechanisms. In fibrotic liver, M-MDSC recruitment correlates with diminished tumor-infiltrating lymphocytes and increased tumorigenicity, as observed in both mouse models and human disease (98). In patients with hepatocellular carcinoma (HCC), the liver is notably enriched for M-MDSCs, and CD33 expression associates with more aggressive tumor features and poorer survival (99). Mechanistically, activated hepatic stellate cells (HSCs) engage monocyte-intrinsic p38 Mitogen-Activated Protein Kinase (MAPK) signaling to drive M-MDSC development and immunosuppression. Consistently, pharmacologic p38 MAPK inhibition disrupts this HSC-M-MDSC circuit and suppresses HCC growth (98).

Metastatic dissemination further highlights the context-dependent roles of monocyte subsets. Metastasis refers to the dissemination of tumor cells to distant organs and their subsequent colonization. “Classical” inflammatory monocytes support metastatic relapse when systemic or local inflammation rises during therapeutic intervention for the primary tumor. For example, in breast cancer, these monocytes are recruited to the tumor site, differentiate into macrophages, and drive metastatic progression by releasing cytokines such as CCL2, which recruit CCR2^+^ inflammatory monocytes to the tumor, where they differentiate into TAMs under the influence of CSF-1 (M-CSF)/CSF-1R signaling and other tumor-associated cytokines. These TAMs promote metastatic progression by creating a pro-tumor environment that supports tumor cell survival, invasion, and dissemination (100). The Chitinase-like protein 3-positive, Lymphocyte antigen 6 complex locus C-high monocyte (Ym1^+^Ly6C^high^) subset, which favors tissue repair, plays a role in this process (101). Consistently, depleting Ly6C^high^ immunoregulatory monocytes suppresses inflammation-driven metastasis, whereas transferring these cells into naïve mice enhances lung metastasis. Additionally, these monocytes express high levels of Matrix Metalloproteinase 9 (MMP-9) and C-X-C chemokine receptor type 4 (CXCR4), and inhibiting either pathway reduces the metastasis-promoting effect (102).

Monocytes and macrophages can promote tumor invasiveness and metastatic potential by engaging adhesion- and stemness-associated pathways, including Cluster of Differentiation 44 (CD44) signaling and interactions with tumor-cell Vascular Cell Adhesion Molecule 1 (VCAM-1), which support cancer cell survival in metastatic niches. Additionally, tumor-intrinsic mechanisms involving ezrin and Phosphoinositide 3-kinase (PI3K) signaling have been implicated in aggressive behavior across multiple cancer types, including breast, prostate, and head-and-neck cancers (103–107). Notably, interrupting monocyte-cancer interactions reverses this invasive phenotype (37). Inflammation-induced monocyte-derived macrophages (CD11b^+^CD11c^+^) increase the efficiency of early metastatic colonization in mouse models. These macrophages secrete hepatocyte growth factor (HGF), which supports tumor cell survival under stress in vitro. Blocking HGF signaling prevents the formation of early micrometastatic lesions that arise during inflammation in vivo. These findings show that HGF-producing macrophages create a pre-metastatic niche and support early survival of tumor cells (108). Moreover, M-MDSCs promote tumor metastasis, coordinating complex interactions between breast cancer cells and stromal cells (109–112).

Conversely, specific monocyte populations can directly inhibit metastasis. Co-culturing undifferentiated monocytes with pancreatic ductal adenocarcinoma (PDAC) cells markedly suppresses invadopodia formation via tissue inhibitor of metalloproteinase-2 (TIMP2) secreted by monocytes. These findings suggest that increasing TIMP2^+^ monocytes at the primary tumor site may block invasion and metastasis (113). In metastatic breast cancer, IFN-γ- producing monocytes are recruited to the lung and activate the TMEM173/STING pathway in neutrophils. This boosts neutrophil killing and prevents the outgrowth of disseminated tumor cells (114). Monocytes induced by IFN-γ (IFN-IMos) also limit lung metastasis by expanding NK cells through an IL-27-dependent mechanism. Forkhead box O1 (FOXO1) and nuclear receptor subfamily 4 group A member 1 (NR4A1) regulate IFN-γ-driven differentiation and the antimetastatic function of IFN-IMos. These findings identify two potential therapeutic targets to improve IFN-γ-based treatment for cancer metastasis (115). Non-classical “patrolling” monocytes, unlike classical monocytes, gather in the lung microvasculature and limit lung metastasis in several mouse models (116).

Together, these findings underscore the functional heterogeneity of monocyte-derived populations in cancer. Although many tumor-associated myeloid cells promote immune suppression and tumor progression, distinct monocyte states can, in specific contexts, exert potent antimetastatic functions, highlighting their therapeutic potential (113, 114, 117).

Together, these findings indicate that monocyte fate decisions in cancer are governed by integrated inflammatory, metabolic, and transcriptional programs rather than linear differentiation pathways.

## 3. Tumor-Driven Reprogramming of Macrophages

Macrophages are professional phagocytes with remarkable plasticity and arise from multiple developmental origins. In cancer, macrophages can derive from bone marrow-produced monocytes that circulate in the bloodstream and are recruited into tissues, where they differentiate into tumor-associated macrophages or undergo programmed cell death (118, 119). In addition, tissue-resident macrophages originate from embryonic progenitors in the yolk sac or fetal liver and persist as long-lived populations with distinct transcriptional and functional identities (120–123). These macrophage populations are widely distributed across tissues and contribute to host defense, immune surveillance, and tissue repair; however, within tumors, they are frequently co-opted to support malignant growth, immune suppression, and remodeling of the tumor microenvironment (124, 125). A central question in tumor immunology is how distinct macrophage populations are reprogrammed by tumor-derived signals to shift from antitumor immune surveillance toward tumor-promoting and immunosuppressive states.

### 3.1 Ontogeny, Polarization, and Functional Plasticity of Macrophages

Macrophage states in tumors reflect both ontogeny (tissue-resident versus monocyte-derived) and dynamic polarization programs induced by local signals. Although the M1/M2 framework has been widely used, tumor-associated macrophages exhibit a continuum of phenotypes, coupled with metabolic remodeling and niche-specific cues. Here, we summarize how the origin and polarization of macrophages intersect to shape macrophage function within the TME and influence whether tumors are controlled or promoted.

Macrophages can be broadly divided into two groups: monocyte-derived macrophages (MDMs) and TRMs. MDMs, derived from the bone marrow, are recruited from the circulation to tissue sites, typically following inflammation. By contrast, TRMs are found in various tissues and arise from embryonic progenitors that seed organs before birth and subsequently self-renew locally. Inflammation and aging can alter tissue macrophage composition by favoring recruitment of bone marrow-derived monocytes, which differentiate and partially replace TRMs in some organs (126). Tissue-resident and monocyte-derived macrophages have distinct origins and consequently display distinct transcriptional programs. As a result, changes in their relative abundance can modify local immune regulation and TME interactions. In tumors, the accumulation of monocyte-derived macrophages often promotes immunosuppressive, pro-angiogenic, and tissue-remodeling functions. This, in part, is mediated by pathways such as CSF-1R signaling, which drive tumor growth, immune evasion, and metastasis. However, the consequences of replacing resident cells with monocyte-derived macrophages are tissue- and context-dependent, and both populations can adopt pro- or antitumor phenotypes depending on the microenvironmental conditions (127–129). Beyond developmental origin, macrophage behavior in tumors is further shaped by functional polarization programs driven by local signals within the TME. Macrophages have classically been categorized into two phenotypes: M1 (“classically activated”) and M2 (“alternatively activated”). M1 macrophages are involved in proinflammatory responses, pathogen clearance, and antitumoral activity. They express CD80 and CD86 cell-surface receptors and secrete IL-1β, tumor necrosis factor α (TNF-α), IL-12, and IL-23. Additionally, M1 macrophages secrete reactive oxygen species (ROS) and have greater antigen-presenting capacity. While these properties support antitumor immunity, they can contribute to chronic inflammation and tissue injury (130).

M2 macrophages are involved in anti-inflammatory responses, tissue repair, angiogenesis, and tumor progression. They express CD206 and CD163 cell-surface receptors, display increased phagocytic activity, express higher levels of Arg-1, and secrete transforming growth factor β (TGF-β), IL-10, and vascular endothelial growth factor (VEGF) (131–134). The secretion of IL-10 and TGF-β by macrophages inhibits T-cell activity, thereby promoting tumor progression rather than clearance. Importantly, macrophage activation is highly plastic, and both pro-inflammatory and anti-inflammatory programs can influence cancer in context-dependent ways. While pro-inflammatory M1 macrophages can support antitumor immunity by enhancing antigen presentation and producing cytotoxic mediators, chronic inflammation can also promote tumor initiation and progression. Persistent inflammation increases mutagenic stress by releasing ROS from myeloid cells, thereby promoting DNA damage and genetic instability. Moreover, inflammatory cytokines, such as TNF-α, activate survival and proliferation pathways, including NF-κB, in tumor and stromal cells. Over time, these processes can favor more aggressive and therapy-resistant tumor cells, helping explain how chronic inflammation promotes tumor progression rather than clearance (135–138).

Macrophage polarization is also tightly linked to metabolic reprogramming. Macrophage activation leads to profound changes in cellular metabolism. M1 activation is characterized by glycolysis and fatty acid synthesis, whereas M2 activation is associated with the tricarboxylic acid cycle, fatty acid oxidation, and glutaminolysis (139). Although widely used, the M1-M2 designation is now recognized as an oversimplification, since it captures only extreme phenotypes along a spectrum of macrophage states. Macrophages and monocytes exhibit considerable diversity and plasticity, enabling them to adapt to various functional states in response to diverse environmental conditions. They exhibit a broader spectrum of activation states rather than the two extreme representatives, M1 and M2. Macrophage complexity is shaped by activation markers, environmental signals, external factors such as substrate stiffness, and epigenetic controls. The continuum of macrophage polarization enables them to adapt to various physiological and pathological conditions. This flexibility complicates classification, as various markers can be expressed across multiple activation states and tissues are exposed to complex stimuli, resulting in mixed macrophage populations. The imbalance in macrophage populations is associated with several immune-related diseases. The continuum is crucial for developing therapeutic strategies that target macrophage polarization to balance inflammation and repair (140, 141).

Within the TME, skewing of this polarization continuum toward M2-like states has profound functional consequences. M2 macrophage polarization promotes tumor-cell proliferation and immune evasion by increasing PD-L1 expression on tumor cells, enhancing secretion of immunosuppressive cytokines such as IL-10 and TGF-β, and driving metabolic changes, including depletion of metabolites such as L-arginine, that inhibit cytotoxic T-cell function (142, 143). Tumor-associated macrophages (TAMs) represent the most abundant immune population within the TME. Despite its antitumor properties, the TME can polarize macrophage phenotypic differentiation and induce tumor-promoting activities, including proliferation, progression, and metastasis (144). Macrophages play numerous roles in tumor progression, and TAMs are crucial at different stages of cancer progression. TAMs induce the expression of cell-surface receptors, cytokine production, and the secretion of growth factors that promote tumor invasion and metastasis (145–148). During the early stages of tumor formation, macrophages control cancer growth through cytotoxicity, phagocytosis, and activation of other innate and adaptive immune responses. As the tumor develops and becomes well established, these macrophages respond to external stimuli, differentiating into phenotypes that suppress immune-cell cytotoxicity and promote tumor survival, proliferation, and progression (149–152). M2 TAMs shape the TME by secreting chemokines that drive immune cell infiltration. M2 TAMs favor immunosuppressive cell subsets, such as regulatory T cells (Treg cells), and inhibit antigen-presenting cells, such as DCs, thereby creating an immunosuppressive TME. M2 TAMs also upregulate receptors involved in the “don’t eat me” signaling pathway, thereby blocking phagocytic uptake and clearance of cancer cells (153). On the other hand, M1 TAMs can participate in a robust adaptive anticancer immune response by enhancing antigen presentation and engaging adaptive immunity.

However, microenvironmental conditions within tumors limit the persistence of these antitumor programs. Hypoxia within the TME reshapes macrophage functional states, suppressing classical M1-associated immune-stimulatory programs while promoting immunosuppressive and tissue-remodeling activities (130). Current TAM-targeted strategies aim to reprogram macrophages toward an M1 pro-inflammatory state or to block “don’t eat me” signaling to restore anticancer activity. It remains unclear which macrophage subsets most strongly drive suppression versus control, in part because clear phenotypic markers are lacking (154, 155).

### 3.2 Tumor-Associated Macrophages as Drivers of Tumor Progression and Therapy Resistance

Tumor-associated macrophages (TAMs) constitute a heterogeneous macrophage compartment that is often skewed toward immunosuppressive, pro-angiogenic, and tissue-remodeling programs. Beyond suppressing antitumor immunity, TAMs directly promote cancer stemness, epithelial-mesenchymal transition, metastatic competence, and therapeutic resistance. In this subsection, we discuss key tumor-promoting functions of TAMs and evidence linking TAM abundance or gene signatures to clinical outcomes.

The presence of TAMs at the tumor site supports cancer progression by promoting M2-like programs that secrete cytokines and growth factors that enhance tumor growth and survival, and TAMs can also contribute to chemotherapy resistance. Mechanistically, TAMs promote drug-resistant cancer stem/initiating cell phenotypes; for example, TAM-derived MFG-E8 activates STAT3 signaling and can cooperate with IL-6 to amplify chemoresistance (156). In addition, TAMs can promote epithelial-to-mesenchymal transition (EMT) programs that confer chemoresistance and metastatic traits, as shown in peritoneal dissemination of pancreatic cancer (157). Clinically, elevated TAM infiltration and/or TAM-associated gene signatures have been associated with poorer therapeutic response and worse outcomes in patient cohorts (158).

Collectively, these tumor-promoting activities reflect a functional shift in macrophages, in which intrinsic antitumor programs are suppressed and replaced by immunosuppressive and pro-tumorigenic states. Macrophages’ primary antitumor function is phagocytosis and clearance of cancer cells; however, the TME reprograms macrophages, impairing their function. TAMs encompass a broad spectrum of M1-M2 macrophages, but their activation and polarization are skewed toward the alternative M2 rather than the classical M1, which is mostly associated with anti-inflammatory responses (159). Macrophage plasticity and heterogeneity are highly diverse and complex; even slight changes in the microenvironment can alter their types, numbers, and functions, thereby affecting their ability to control tumor development and response to therapy (160). The TME’s ability to influence monocyte/macrophage function and phenotype depends on its composition, which is directly influenced by tumor stage, site, and type (161). Notably, M2-polarized TAMs are mostly localized in the hypoxic areas of the TME (162). Overall, tumor-associated immune cells, especially TAMs, have been associated with poor prognosis and reduced patient survival (163–165). TAMs release immunosuppressive factors that inhibit T-cell proliferation, thereby blocking activation of the adaptive immune response, which is crucial for controlling tumor growth and clearance (166, 167). Collectively, these studies position TAM abundance and functional skewing as key determinants of therapeutic resistance across tumor types.

### 3.3 Macrophage Receptors and Immune Checkpoints Shaping the Tumor Microenvironment

Macrophage behavior in tumors is shaped not only by soluble cues but also by receptor-ligand interactions that regulate recruitment, activation, antigen presentation, and immune suppression. Tumor-associated macrophages frequently upregulate inhibitory receptors and checkpoint ligands while retaining activating pathways that can be therapeutically leveraged to reprogram. Here, we highlight key macrophage receptors in the TME and discuss how targeting these pathways can restore phagocytosis and antitumor immunity.

During tumor formation, innate immune cells are recruited to the tumor site via chemokines and receptor-ligand-mediated signaling, where they recognize tumor cells and initiate antitumor responses. However, these same immune cells can be reprogrammed to drive tumor-related inflammation, promoting progression rather than control and clearance (45, 168). Monocyte development and differentiation in the bone marrow are governed by CSF1R signaling through its ligands CSF-1 and IL-34 (169). Once in circulation, chemokine receptors such as CX3CR1 guide monocyte trafficking into tumors, where they phagocytose cancer cells and associated material (116). Within the TME, particularly in hypoxic niches, monocytes/macrophages often polarize toward an M2-like phenotype, and CD209 expression is observed on M2-skewed TAMs in hypoxic regions (170). In addition, M2 TAMs express CD204, which is correlated with high CCL2 levels and a tumor-promoting microenvironment (171, 172).

Most TAMs induce immunosuppressive signaling by expressing inhibitory receptors, such as the non-classical MHC-I molecules HLA-E and HLA-G, and the immune checkpoint ligands PD-L1, PD-L2, CD80, and CD86 (144). M2 TAMs express PD-L1 to support cancer cells’ immune evasion (142). M2-skewed TAMs commonly express high levels of PD-L1 (Table 1). Functionally, PD-L1 expressed on TAMs engages PD-1 on T cells, suppressing activation and cytotoxicity, while CD80/CD86 binding to cytotoxic T-lymphocyte-associated protein 4 (CTLA-4) further inhibits T-cell responses (Table 1) (173). In addition, TAMs that release IL-1β induce upregulation of PD-L1 expression on tumor cells, contributing to immune escape (174). TAMs help maintain an immunosuppressive environment by secreting IL-10 and TGF-β, which inhibit both CD4 and CD8 T cells and promote the differentiation of regulatory T (Treg) cells. TAMs recruit Treg cells within the TME through the production of numerous chemokines and by inhibiting T-cell cytotoxicity, either by depleting L-arginine via Arg-1 release or by depleting tryptophan via indoleamine 2,3-dioxygenase (IDO) expression (175). Tregs are immunosuppressive lymphocytes that promote tumor progression by limiting antitumor immune responses within the tumor microenvironment. They are actively recruited to tumor sites by chemokines such as CCL1, CCL22, and CCL28, or can be induced from conventional CD4^+^ T helper cells in the presence of TGF-β. Once established in the TME, Tregs suppress effector T-cell activity, promote immune tolerance, and contribute to tumor immune evasion (176–180).

PD-L1 expression is often increased in M2-like tumor-associated macrophages (TAMs), particularly CD163^+^ macrophages. Nasopharyngeal carcinoma, prostate cancer, and cervical carcinoma show a positive correlation between CD163^+^ M2-type macrophage infiltration and PD-L1 expression. In cervical cancer, M2-type TAMs upregulate PD-L1 expression by activating the PI3K/AKT pathway. The interaction between PD-L1 on TAMs and PD-1 on T cells inhibits T cells, thereby promoting immune evasion. This is a key immunosuppressive mechanism by which tumors progress. PD-1/PD-L1 checkpoint inhibitors restore antitumor T-cell activity by interrupting inhibitory signals driven by PD-L1 on tumor cells and immune cells, including tumor-associated macrophages. However, patient responses vary widely, and PD-L1-expressing myeloid cells can contribute to an immunosuppressive TME, potentially limiting therapeutic efficacy in some patients. This has led to the development of combination approaches that target TAM recruitment or polarization to improve responses to checkpoint blockade. For example, inhibition of CSF1R has been shown to reprogram TAMs toward a less immunosuppressive phenotype and enhance the efficacy of anti-PD-1 therapy in colorectal cancer models. Together, these findings support combining myeloid-targeted therapies with PD-1/PD-L1 blockade to achieve more durable and effective clinical responses (181–185). CD40, a member of the TNF receptor superfamily, is expressed on DCs, B cells, and macrophages (including TAMs) (Table 1). When CD40 binds its ligand CD40L, it triggers tumor-specific T-cell immune responses. Consistent with this, agonistic anti-CD40 antibodies, designed to mimic CD40L binding to CD40, can inhibit tumor growth by activating DCs and repolarizing TAMs toward an M1 phenotype (186, 187). The CD40/CD40L axis leads to DC activation and upregulates co-stimulatory molecules (CD80, CD86) and MHC molecules (Table 1). Interestingly, CD40 agonists have been shown to elicit tumor-specific immune responses and, in some malignancies, to directly induce tumor-cell death (188–192). Therapeutically, CD40 can be targeted to induce pro-inflammatory cytokines and activate cytotoxic T cells (187, 193).

Recognition of tumor-derived danger signals or dsRNA by TLR3 skews macrophages toward an M1 phenotype while suppressing M2 features (Table 1). Activation of TLRs on macrophages promotes M1 polarization, the release of pro-inflammatory cytokines such as IL-1β, IL-6, IL-12, and TNF-α, and the activation of the type I interferon response. Through IL-12-driven activation of NK and T cells, this pathway can also promote IFN-γ-associated Th1 immunity. This response involves the production of pro-inflammatory cytokines, which enhance antitumor immunity through mechanisms such as antigen presentation, nitric oxide production, and the activation of Th1-polarized and cytotoxic immune cells. However, when TLR signaling persists or becomes excessive, inflammatory cytokines such as IL-6 and IL-1β are produced at elevated levels. These cytokines activate signaling pathways, most notably the JAK/STAT3 pathway, in both tumor cells and surrounding stromal cells. Activation of this pathway supports tumor cell survival and proliferation and is associated with chemotherapy resistance. Additionally, endothelial cell activation induces VEGF expression, promoting angiogenesis and altering vascular structure, thereby facilitating tumor invasion and metastatic spread. Chronic inflammation also affects the extracellular matrix by inducing matrix-degrading proteases, gradually removing physical barriers that would otherwise limit tumor cell invasion. Sustained inflammatory activity further contributes to hypoxia in the TME. Under hypoxic conditions, hypoxia-inducible factors such as HIF-1α and HIF-2α drive transcriptional programs associated with angiogenesis, immune evasion, and metabolic adaptation. These hypoxia-driven changes favor macrophage recruitment and reprogramming toward M2-like phenotypes, thereby restoring an immunosuppressive environment that ultimately supports continued tumor growth and progression (194–203). TLR7 and TLR7/8 agonists, such as imiquimod (approved for superficial basal cell carcinoma and actinic keratosis) and resiquimod, have demonstrated autophagy-mediated tumor-cell death in preclinical models (204). Consistent with this, TLR7/8 agonists have been used to reprogram TAMs from an M2 to an M1 phenotype (205). In contrast, chronic or sustained TLR4 signaling in TAMs can be pro-tumorigenic by driving IL-10 production and immunosuppressive polarization (206).

The expression of CD137 has attracted significant interest because of its dual role in inducing pro-inflammatory cytokines and regulating IL-10 secretion (Table 1). Activation of CD137 signaling enhances the function of antigen-presenting cells (APCs), thereby inducing T-cell activation and contributing to tumor control (207). CD137 (4-1BB) is a TNF receptor expressed on immune cells, including NK cells; signaling through the CD137-CD137 ligand axis is vital for CD8+ cytotoxic T lymphocyte activation, proliferation, and differentiation. Agonistic anti-CD137 mAbs enhance NK-cell-mediated ADCC and potentiate the antitumor efficacy of anti-CD20 mAbs (e.g., rituximab) by further activating NK cells in lymphoma (208–210). Similarly, interleukin-12 (IL-12) produced by antigen-presenting cells induces interferon-γ (IFN-γ) production by T and NK cells, and IFN-γ, in turn, upregulates MHC class I and II, strengthening T-cell activation (211, 212).

Additional receptors important for TAM biology and function include CD206, MARCO, folate receptor-β (FRβ/FOLR2), and epidermal growth factor receptor (EGFR). TAMs expressing CD206^+^ are typically immunosuppressive and can be therapeutically targeted (213) (Table 1). CD206, a C-type lectin receptor of the mannose receptor family, is expressed on M2 macrophages, and blocking or depleting CD206^+^ TAMs can shift the compartment toward a more pro-inflammatory M1 phenotype (214). Similarly, targeting the scavenger receptor MARCO polarizes M2-polarized TAMs toward a pro-inflammatory and antitumor state (215). FRβ/FOLR2, selectively expressed on M2-like TAMs, promotes immunosuppression. High FRβ activity is associated with reduced CD8^+^ T-cell infiltration and tumor growth (216, 217). In addition, M2-like TAMs produce epidermal growth factor (EGF), which binds EGFR on tumor cells, driving proliferation, invasion, and metastasis (218).

### 3.4 Cytokine-Chemokine Networks Governing TAM Recruitment and Polarization

Recruitment and functional polarization of TAMs are coordinated by interconnected cytokine and chemokine networks generated by tumor cells, stromal elements, and immune populations. These circuits not only control monocyte trafficking and macrophage positioning within hypoxic or invasive niches, but also reinforce immunosuppressive programs that drive metastasis and therapy resistance. In this subsection, we outline major chemokine axes and cytokine loops that shape TAM accumulation and phenotype, highlighting context-dependent effects and therapeutic opportunities.

Many chemokines have been linked to the recruitment of macrophages to the tumor site. Chemokine (C-C motif) ligand 2 (CCL2), CCL5, chemokine (C-X-C motif) ligand 12 (CXCL12), and chemokine (C-X3-C motif) ligand 1 (CX3CL1) are known tumor-promoting chemokines that primarily recruit macrophages to the TME (219–222) (Table 2). CCL1 is frequently upregulated in cervical squamous cell carcinoma and other squamous carcinomas, such as esophageal squamous cell carcinoma, where CCL1-CCR8 signaling contributes to a pro-tumorigenic microenvironment and promotes cancer progression and metastasis (223, 224) (Table 2). Expression of CCL1 and its receptor CCR8 by M2 macrophages promotes tumorigenesis (225–227). In addition, secretion of CCL3 by mantle cell lymphoma cells promotes M2 polarization of macrophages via C-C chemokine receptor 1 (CCR1) signaling, thereby enhancing cancer invasion and immune escape (228).

The interaction between CCL2 and its receptor, CCR2, is triggered by pro-inflammatory signals and recruits macrophages (Table 2). Local cytokines then skew differentiation toward M2-like TAMs, which release proteases that sustain inflammation and facilitate invasion (229). In breast cancer, CCR2-expressing monocytes, once recruited to mammary tumors, are well known to promote tumor growth and metastasis. However, CCR2 is also expressed on cancer cells, where it can modulate tumor growth and immune responses in a context-dependent manner. In tumor cells, CCR2 signaling has been reported to primarily influence immune evasion rather than directly promote monocyte recruitment, including by regulating PD-L1 expression and modulating the activity of cytotoxic T cells and CD103^+^ cross-presenting dendritic cells. Consistent with this complexity, several studies indicate that CCL2-expressing monocytes or macrophages can exert antitumor effects under specific conditions, including enhancing antigen presentation, promoting cytotoxic T-cell responses, and suppressing tumor growth. These opposing outcomes suggest that the effects of the CCL2/CCR2 axis depend on the activation state of monocytes and macrophages, tumor stage, and the microenvironmental context. Accordingly, phenotypes observed in Ccr2^−^/^−^ models may reflect complex, cell-intrinsic effects beyond monocyte recruitment alone, supporting the notion that the CCL2/CCR2 axis is a key immune-modulatory pathway in cancer whose function is highly context dependent and can be exploited by both immune cells and cancer cells to shape tumor immunity (230–232).

In addition to CCR2-dependent signaling, CCL2 can engage alternative chemokine pathways that further regulate monocyte retention and TAM positioning within tumors. For example, CCL2 secretion triggers a chemokine cascade, including CCL3 signaling via CCR1, which promotes monocyte retention and supports tumor growth and metastasis. Consequently, blocking CCL2-CCR1 signaling inhibits the recruitment of inflammatory monocytes. TGF-β also upregulates CXCR4 expression in TAMs, leading to CXCR4-positive TAMs migrating toward CXCL12-producing tumors, thereby promoting cancer cell intravasation (34, 39, 233). In melanoma and bladder cancer, tumor cell-derived CCL2 interacts with CCR2 on TAMs, inducing macrophage recruitment, infiltration, and polarization toward an M2 phenotype (234–236). IL-3 and CCL2 together support macrophage differentiation, growth, and cytokine secretion. Interestingly, CCL2 and CXCL10 can also facilitate T-cell migration to the tumor site when TAMs are depleted (237). However, in most contexts, CCL2 promotes M2 polarization and tumor metastasis (238, 239). Recruitment of regulatory T cells (Tregs) is also regulated by CCL2, IL-10, and the CXCL12/CXCR4 axis. CCL2 additionally cooperates with IL-6 to promote the differentiation and survival of myeloid cells (240). Clinical evidence shows a correlation between high CCL2 expression and increased infiltration of CD68^+^/CCR2^+^ macrophages in tumors, which is associated with high proliferative activity and reduced survival in patients (241, 242). Importantly, inhibition of CCL2 (e.g., with arsenic trioxide) has been shown to reduce M2 polarization (243). The expression of CCL5 and its receptor, CCR5, facilitates macrophage migration to the tumor site (244) (Table 2). Breast cancer often shows high CCL5 expression, which supports monocyte recruitment to the tumor (244, 245). The recruited cells differentiate and express high levels of CCL3, CCL4, and CCL5, thereby recruiting NK cells to mediate tumor-cell killing (38). M1 macrophages express high levels of CXCL9, CXCL10, and CCL5, which are associated with antitumor activity (246). However, CCL5 secreted by myofibroblasts in the TME binds to CCR5 on TAMs, activating pathways that promote M2 polarization and induce CCL18 secretion, thereby supporting tumor growth (247).

Hypoxia in the TME activates HIF-dependent programs that can increase pro-tumor mediators such as TGF-β, CXCL12, and CCL5 and modulate the CX3CL1/CX3CR1 axis, thereby promoting tumor progression (258). TGF-β stimulates monocyte chemotaxis into the TME and induces immunosuppression, thereby favoring tumor growth and metastasis (249). TGF-β exerts a dual role: tumor-suppressive in early stages (by limiting proliferation) but, at later stages, pro-tumorigenic by expanding Tregs and promoting M2 polarization, which supports metastasis (250–256). IL-10 and TGF-β favor Treg activation over Th1 responses. TAMs secrete CCL22 (and, in some contexts, CCL17) to recruit CCR4^+^ regulatory T cells, while CCL18 attracts naïve T lymphocytes and promotes Th2-biased and immunosuppressive immune responses (257, 258) (Table 2). Consistent with this, elevated CCL22 correlates with tumor progression in breast cancer patients (259). CXCL12 guides TAMs to hypoxic niches within the TME and drives M2 polarization (for example, via CD163 expression), while CX3CL1 also contributes to TAM recruitment and intratumoral infiltration (260, 261).

Interleukins produced in the TME reshape TAM function, activating cytokine circuits that promote resistance to therapy, proliferation, invasion, and metastasis (Table 2). For example, mixed-phenotype TAMs (M1/M2-like macrophages) can express high Arg-1 and secrete IL-10, together contributing to a TME that promotes enhanced cancer cell migration and invasiveness (262–264). Tumor malignancy is further promoted by IL-10 secreted from M2 macrophages, which suppresses antitumor immunity (Table 2). In hypoxic regions of the TME, TAMs secrete CXCL8 and engage CXCR1/2 signaling pathways, thereby enhancing IL-10 production and reinforcing M2 polarization (265, 266). Some cancer cells also release IL-32, which induces a phagocytic, M2-like phenotype in TAMs. These cells secrete IL-10, which facilitates metastasis (267). Both TAMs and cancer cells secrete IL-6 and IL-10, establishing reciprocal signaling that drives cancer progression (Table 2). IL-10 not only promotes macrophage differentiation but also suppresses DC development, further impairing adaptive immunity. The presence of IL-10 promotes macrophage differentiation while suppressing DCs (268). At the tumor site, TAMs contribute to immune evasion and tumor growth by producing immunosuppressive mediators such as CCL17/CCL22, TGF-β, and IL-10; generating prostaglandin E2 (PGE2); expressing high levels of Arg-1; and releasing immune-regulatory lectins, including galectin-1 and galectin-9 (269–272). While M1 TAMs are generally considered antitumor, their secretion of IL-6 can paradoxically activate signaling pathways such as NF-κB and STAT3 within the TME, thereby supporting tumor progression and metastasis (273–275) (Table 2). In most cancers, however, IL-6 predominantly acts through M2 TAMs, promoting polarization, metastatic spread, and interaction with IL-6R on cancer cells (276). M2 macrophages also secrete IL-6 and respond to IL-4, driving metastasis and resistance to cancer therapies (277). Furthermore, tumor-derived extracellular vesicles (EVs) carrying inflammatory mediators can shape macrophage polarization. For example, EV-associated IL-32 released by tumor cells promotes M2-like macrophage polarization (267), while macrophage-derived IL-6 activates IL-6/STAT3 signaling in pancreatic cancer cells, thereby favoring tumor progression (278). Finally, the combined release of IL-6 and IL-10 by TAMs activates B7-H4 via STAT3 signaling, thereby inhibiting cytotoxic T-cell responses and promoting immune escape (279–282).

Other cytokines and chemokines implicated in cancer include IL-33, IL-34, M-CSF (CSF1), the M-CSF receptor (CSF1R), CCL3, CCL4, CCL7, CCL8, vascular endothelial growth factor (VEGF), and platelet-derived growth factor (PDGF) (Table 2). In pancreatic cancer, IL-33 induces M2 polarization, thereby enhancing cancer cell migration and invasion (283). Tumor-derived EVs containing immunoregulatory cargo can drive M2-like macrophage polarization (284). CSF1/CSF1R signaling sustains pro-tumor TAM programs, and its blockade reprograms macrophages toward an M1-like phenotype (285). CSF1R signaling supports TAM recruitment and survival, and inhibitors reduce TAM abundance at tumor sites and can suppress tumor growth (286). In addition, chemokines and cytokines such as CCL3, CCL4, CCL7, and CCL8, as well as M-CSF and IL-10, and growth factors including VEGF and PDGF, promote monocyte/macrophage recruitment to tumors (146, 287). These interconnected cytokine-chemokine circuits reinforce macrophage recruitment, spatial positioning, and immunosuppressive polarization, thereby stabilizing tumor-supportive niches.

## 4. Targeting myeloid cells in cancer: Depletion, Recruitment Blockade, Reprogramming

Building on these mechanistic insights, the limitations of T-cell-directed immunotherapies across many tumor types underscore the importance of tumor-intrinsic and microenvironmental mechanisms of immune resistance. Monocytes, tumor-associated macrophages, and myeloid-derived suppressor cells collectively define a myeloid immune landscape that shapes tumor immune evasion and therapeutic outcomes. Tumor-associated monocytes and macrophages are central architects of immune suppression and therapy failure, making the myeloid compartment an attractive and increasingly tractable therapeutic target. In this section, we summarize myeloid-directed strategies across three complementary modalities, including depletion, recruitment blockade, and functional reprogramming, and highlight emerging combination approaches and clinical challenges.

Cancer is a leading public health challenge worldwide, and its incidence and mortality continue to rise despite advances in therapies and surgical interventions (288, 289). Developing effective therapies depends on understanding the immune checkpoints that suppress cancer growth. Studying the pathways involved in tumor control can help identify new therapeutic targets. Although several immune T-cell checkpoint inhibitors have been approved by the FDA, only about one-quarter of patients respond, highlighting the need for alternative strategies, such as blocking other inhibitory pathways or targeting co-stimulatory receptors (290, 291).

The diverse roles of TAMs in the TME influence tumor survival, progression, and responses to therapy (292). Because M2 macrophages are major drivers of tumor progression, strategies that block their recruitment, deplete them from the TME, or redirect monocyte differentiation toward an M1 phenotype could provide therapeutic benefits (293). Approaches that promote M1 polarization while preventing M2 polarization are promising strategies for restraining tumor progression. Likewise, designing agents that selectively deplete existing TAMs, particularly M2 macrophages, may yield additional therapeutic opportunities.

**Figure 2.**
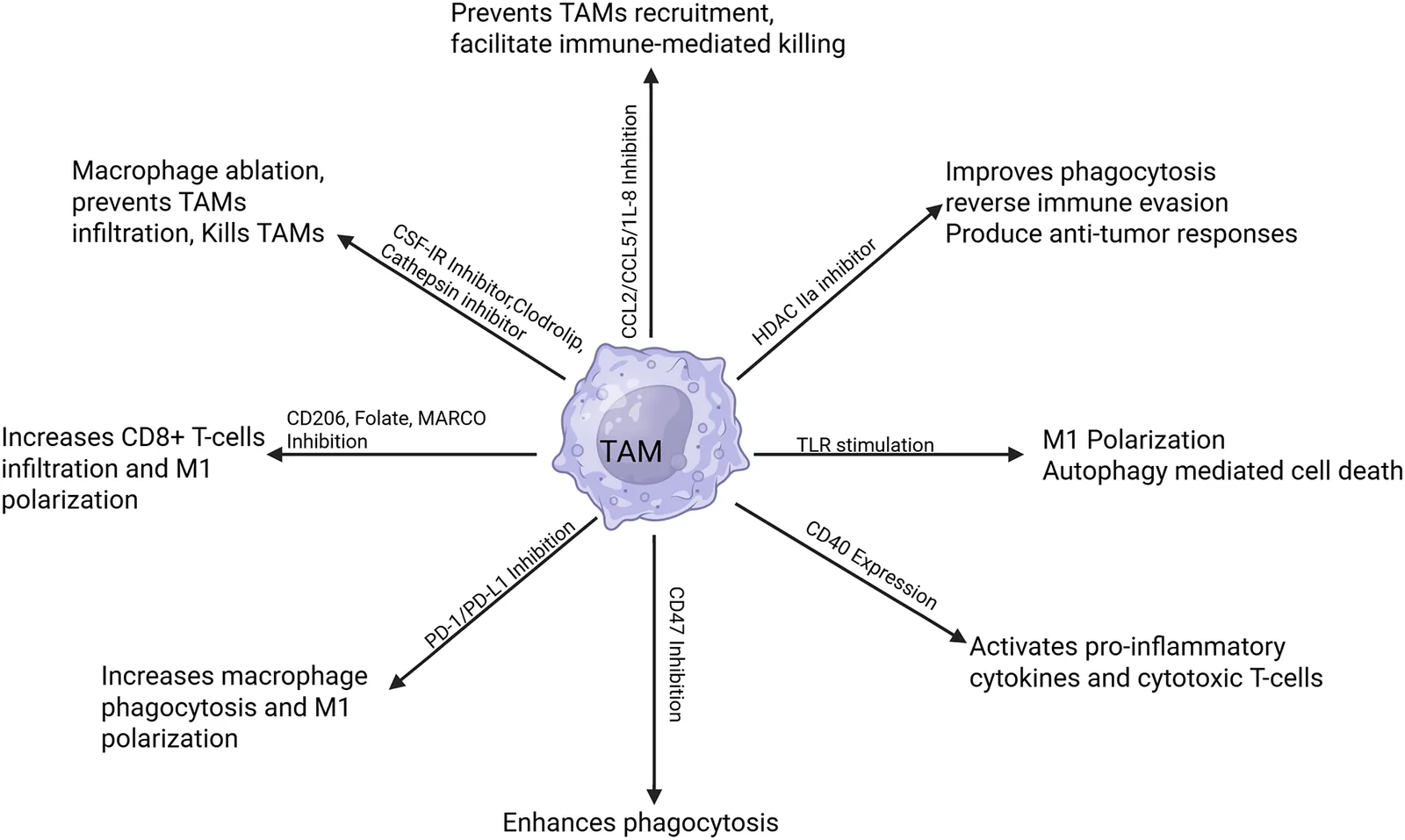
Immunotherapeutic strategies targeting tumor-associated monocytes and macrophages. Schematic overview of therapeutic approaches designed to counter the pro-tumor functions of tumor-associated macrophages (TAMs) within the tumor microenvironment (TME). Strategies include: inhibition of monocyte recruitment using antagonists of chemokine (C-C motif) ligand 2 (CCL2)/C-C chemokine receptor 2 (CCR2), chemokine (C-C motif) ligand 5 (CCL5)/C-C chemokine receptor 5 (CCR5), and interleukin-8 (IL-8)/C-X-C chemokine receptor 1 or 2 (CXCR1/2); functional reprogramming of TAMs toward an M1-like phenotype using Toll-like receptor (TLR) agonists, cluster of differentiation 40 (CD40) agonists, and class IIa histone deacetylase (HDAC) inhibitors; enhancement of phagocytosis and antigen presentation through blockade of the signal regulatory protein-α (SIRPα)-cluster of differentiation 47 (CD47) axis and the programmed cell death protein-1 (PD-1)/programmed death-ligand 1 (PD-L1) immune checkpoint; relief of immunosuppression and improved cytotoxic T-cell infiltration by targeting macrophage receptor with collagenous structure (MARCO), the mannose receptor (CD206), and folate receptor-β (FOLR2); and depletion of TAMs or limitation of their survival using colony-stimulating factor-1 receptor (CSF1R) inhibitors, clodronate-loaded liposomes (clodrolip), and cathepsin inhibitors.

TAM-directed strategies fall into three complementary categories: depletion, recruitment blockade, and functional reprogramming. Depleting TAMs, particularly M2 macrophages, can be achieved with cathepsin inhibitors, which target lysosomal cathepsins that promote M2 polarization and tumor growth. Clodronate liposomes (Clodrolip) and CSF-1R inhibitors are also effective at depleting TAMs. Clodronate-loaded liposomes have demonstrated efficacy in ablating macrophages, preventing infiltration, and providing antitumor effects against metastasis, while CSF-1R inhibitors reduce TAM survival within the TME (294–299) (Fig. 2). To limit additional TAM recruitment to the tumor site, preclinical studies show that blocking chemokine axes effectively reduces monocyte migration. The CCL5/CCR5 axis is a key driver of monocyte recruitment into the TME, and its inhibition suppresses tumor growth and metastasis. Likewise, tumor-derived IL-8 recruits monocytes, and antibody-mediated neutralization of IL-8 can diminish recruitment and facilitate immune-mediated killing (300–303). Because CCL2/CCR2 and CXCL12/CXCR4 are major regulators of monocyte trafficking, their inhibition may further promote CD8^+^ T-cell infiltration and enhance antitumor responses (304–307) (Fig. 2).

Reprogramming TAMs toward an M1 phenotype is also a promising therapeutic avenue. Because M1 macrophages exhibit antitumor activity, enforcing this phenotype with interferon regulatory factor 5 (IRF5) can increase M1 macrophages in the TME (308–310). Epigenetic interventions, including histone acetylation and methylation, offer another therapeutic opportunity. The class IIa histone deacetylase inhibitor TMP195 reprograms monocytes/TAMs into highly phagocytic cells that recruit CD8^+^ T cells (311), and its combination with DNA methyltransferase inhibitors has been shown to reverse immune evasion by reprogramming TAMs. More broadly, HDAC inhibition enhances T-cell responses (312) (Fig. 2). In addition, because M-CSF and IL-34 promote M2 polarization via CSF1R, blocking this pathway can favor M1 polarization and strengthen antitumor immunity (313, 314). Finally, elevated IL-6 is associated with poor therapeutic response and M2 skewing; therefore, inhibiting IL-6-driven pathways may reduce M2 polarization and improve treatment outcomes (315).

As a complementary strategy to cytokine blockade, TLRs can be activated to enhance tumor recognition by upregulating M1-associated receptors, such as CD80/CD86, and downregulating M2 markers. In the presence of IFN-γ, TLR agonists preferentially drive M1 over M2 polarization. Agonists for TLR3, TLR4, TLR7, and TLR8 have been developed to reprogram M2 macrophages toward an M1 phenotype, thereby inducing autophagy-mediated tumor cell death (316–318) (Fig. 2). The cell-surface receptor CD40 has also been targeted therapeutically, as its activation induces pro-inflammatory cytokines and activates cytotoxic T cells to mount antitumor responses. Stimulating CD40 signaling in macrophages or other antigen-presenting cells (APCs) by binding to its natural ligand, CD40L, can reduce tumor growth (193, 319) (Fig. 2). CD47, often highly expressed on tumor cells, binds SIRPα on macrophages to deliver a “don’t-eat-me” signal that blocks phagocytosis of cancer cells and supports immune evasion. Antibodies targeting CD47 or SIRPα can enhance tumor-cell phagocytosis and reduce tumor growth and metastasis (320–324) (Fig. 2). Although CD47 has emerged as an attractive innate immune checkpoint, its clinical translation remains challenging. Most CD47-targeting agents have shown limited clinical success, largely because CD47 is ubiquitously expressed on normal cells, including red blood cells and platelets, leading to on-target hematologic toxicities. These limitations have prompted the development of next-generation strategies to improve tumor selectivity and minimize systemic toxicity (325–329). The mannose receptor (CD206), MARCO, and the folate receptor are additional targets because their expression is typically linked to M2-TAMs. Blocking these receptors can promote pro-inflammatory cytokine production, increase CD8^+^ T-cell infiltration, raise the M1 macrophage ratio, and decrease tumor growth (215–217) (Fig. 2).

Finally, stimulating macrophage phagocytic capacity through immune checkpoint blockade can further enhance their function within the TME. PD-L1, expressed on tumor and immune cells, binds to PD-1 on T cells, inducing immune tolerance. PD-L1, expressed on tumor and immune cells, binds PD-1 on both T cells and tumor-associated macrophages, thereby suppressing immune activation and macrophage phagocytosis. Blocking the PD-1/PD-L1 axis increases macrophage-mediated phagocytosis, both directly and indirectly, in part by enhancing IFN-γ secretion, which, in turn, promotes the expression of costimulatory molecules and pro-inflammatory mediators such as iNOS, CD40, and MHC II. These downstream signals enhance macrophage activation, survival, and functional remodeling in a T-cell-dependent manner, thereby contributing to improved control of tumor growth (328–331). Although PD-1/PD-L1 therapy shows promise, with monoclonal antibodies against ipilimumab (a CTLA-4 inhibitor) and nivolumab (a PD-1/PD-L1 inhibitor) now approved for antitumor activity, further improvements are needed, as only a small percentage of patients experience prolonged survival (332–336).

Despite numerous suggestions in the literature, few successes have emerged, and those that have are limited in scope. The CSF1R inhibitor pexidartinib and the anti-CSF1R antibody RG7155 (emactuzumab) are effective in conditions characterized by CSF1 overexpression and have shown a striking reduction in CSF1R^+^ CD163^+^ macrophages in tissues. A limitation of these therapies is that they often act as single agents, limiting their efficacy in solid tumors. Preclinical animal models suggest that even with a combination of anti-VEGF and taxane chemotherapy, there is a significant reduction in tumor burden in ovarian cancer models. Additionally, PD-1/PD-L1 blockade-based combination therapies have yielded better outcomes, including increased CD8^+^ T-cell infiltration and reduced tumor progression, compared to single-target therapies. A triple therapy of ipilimumab, nivolumab, and trabectedin in a phase II trial shows acceptable safety and preliminary clinical activity in patients with soft-tissue carcinoma. Lastly, the combination of the anti-CSF1R mAb emactuzumab and the PD-L1 mAb atezolizumab increased CD8^+^ T-cell infiltration, concomitant with a reduction in tumor-associated macrophages (337–342). The recruitment of myeloid cells, particularly immunosuppressive cells, into the TME is mediated by cytokines and chemokines. Therefore, strategies have been designed to inhibit chemokines, chemokine receptors, and signaling checkpoints, such as PI3K-γ, LILRB4, and JAK1/2, to block myeloid cell recruitment, and these strategies have shown efficacy in mouse models and early clinical studies. In a phase I clinical trial, the combination of the CCR2 inhibitor PF-04136309 with standard chemotherapy was shown to be safe and to inhibit TAM recruitment, thereby restricting tumor growth in pancreatic ductal adenocarcinoma. Similarly, Carlumab, a human anti-CCL2 IgG1κ monoclonal antibody, was well tolerated, with evidence of transient suppression of free CCL2 and of antitumor activity (343–348).

Recent clinical trials have also focused on TLR agonists to reprogram and restore M1 macrophages. A TLR9 agonist, MGN1703, has shown promising results in small-cell lung cancer. However, conclusions cannot be drawn due to the limited sample sizes and patient numbers. TLR4 agonists, such as GSK1795091, are under clinical investigation, although their therapeutic efficacy has not yet been fully established. Other reprogramming strategies involve inhibiting the PI3K/AKT signaling pathway, which is implicated in cancer pathogenesis, and have shown promise when PI3Kδ/γ is blocked with the PI3Kδ/γ inhibitor tenalisib (RP6530), either as a single agent or in combination with other immunotherapies, in patients with T-cell lymphoma. Additionally, a mouse model of breast and lung cancer demonstrates that loading mannosylated dual pH-responsive nanoparticles with siRNAs targeting placental growth factor and VEGF silences these genes, suppresses tumor proliferation, and inhibits metastasis (349–353).

## 5. Discussion

**Figure 3.**
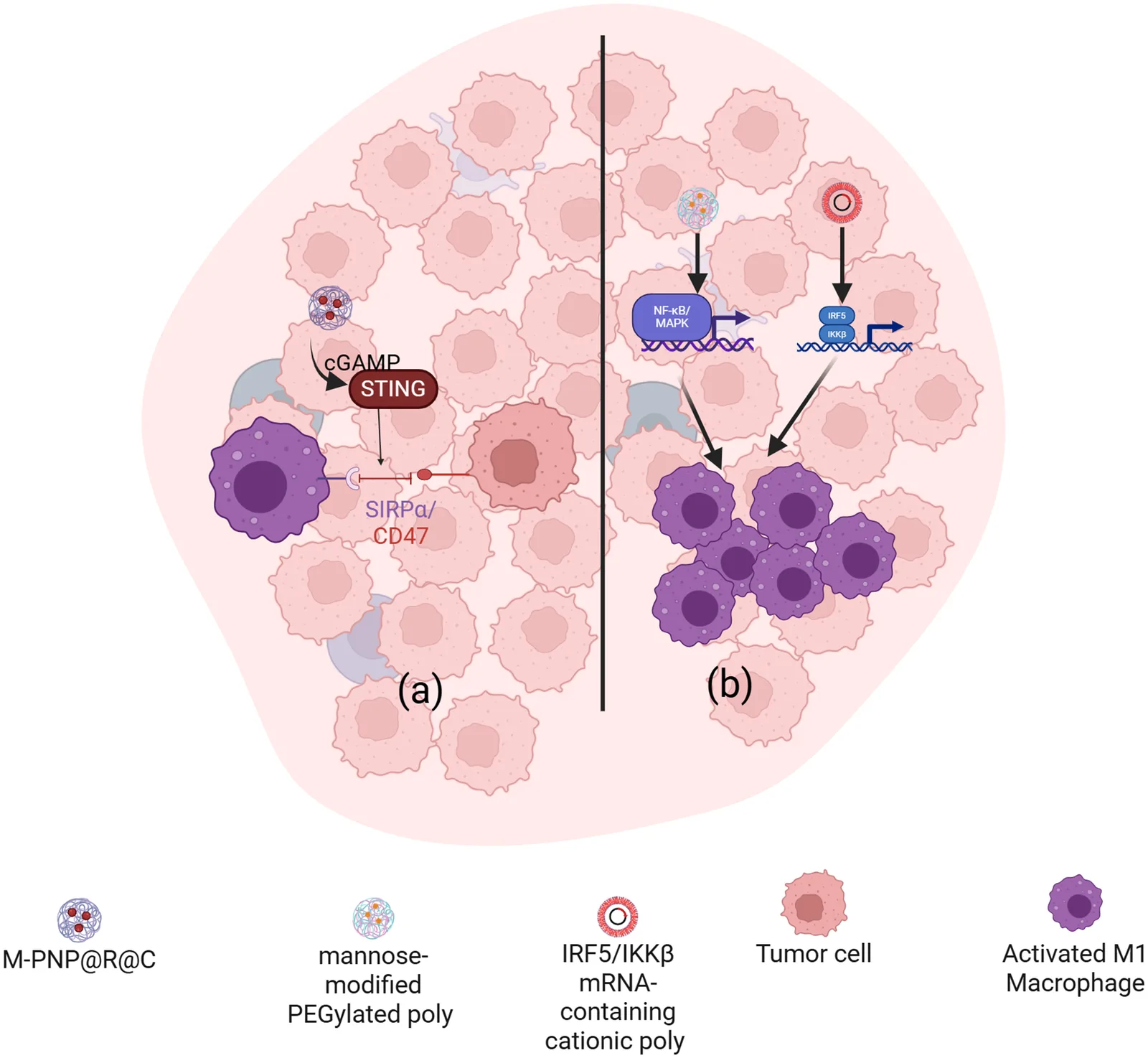
Conceptual designs for macrophage-directed nanoparticle therapies. M-PNP@R@C nanoparticles co-formulated with a Toll-like receptor (TLR) 7/8 agonist and 2′,3′-cyclic guanosine monophosphate-adenosine monophosphate (cGAMP), designed to activate the stimulator of interferon genes (STING) pathway in macrophages. STING activation reduces signal regulatory protein-α (SIRPα) expression, thereby alleviating the cluster of differentiation 47 (CD47)-SIRPα “don’t-eat-me” checkpoint and enhancing macrophage-mediated phagocytosis. Mannose-targeted polymeric nanocarriers consisting of mannose-modified, polyethylene glycol (PEG)-coated cationic polymers engineered to deliver either adjuvant/polyplex cargo that activates nuclear factor κB (NF-κB) and mitogen-activated protein kinase (MAPK) signaling pathways, or messenger RNA (mRNA) encoding interferon regulatory factor 5 (IRF5) and inhibitor of nuclear factor κB kinase subunit β (IKKβ). Both strategies aim to promote M1-like macrophage activation and functional reprogramming in the tumor microenvironment (TME).

The TME contains immune cells that are often reprogrammed by local signals to promote tumor growth. The TME recruits a large proportion of monocytes/macrophages, which are reprogrammed toward M2 polarization. TAMs play a significant role in tumor progression; therefore, understanding how they are recruited, polarized, and respond to signals can guide the development of novel therapies for patients. A deeper understanding of the roles of receptors, cytokines, and chemokines in shaping TAMs will inform strategies to deplete or reprogram them to improve cancer treatment. Multiple studies show that monocytes/macrophages can defend the body against tumors. In the TME, monocytes are recruited as the first line of defense and express receptors and secrete cytokines/chemokines that trigger downstream cytotoxic CD8^+^ T-cell activity to control tumor progression and metastasis. However, the TME can reprogram monocytes and macrophages to shift their polarization toward suppression of antitumor activity and support of tumor growth (354, 355). Therefore, nanoplatform-based therapies that boost M1 macrophage activity while preventing M2 polarization are highly promising (Fig. 3).

In this regard, a mannose-modified, pH-sensitive nanoplatform co-encapsulating resiquimod (R848), a TLR7/8 agonist, and 2’,3’-cyclic GMP-AMP (cGAMP), named M-PNP@R@C, repolarizes TAMs from an M2 to an M1 state. This intervention activates STING signaling, downregulates SIRPα, and blocks its interaction with CD47, thereby inhibiting the CD47-SIRPα axis. Because SIRPα suppresses phagocytosis, blocking this pathway reduces immune evasion (Fig. 3). Combination therapy with anti-CD47 and PD-L1 antibodies has shown higher survival rates and reduced metastasis in melanoma-grafted in vivo models (356). Additional nanotechnologies have been developed to reprogram M2 phenotypes, inhibit M2 recruitment, and deplete TAMs. For depletion, nanomaterials include a clodronate-cationic nanoliposome nanogel that depletes TAMs (357). Cell-derived vesicles from M1 macrophages carrying cGAMP can activate STING and disrupt the CD47-SIRPα axis (356, 358), and azide-modified exosomes from M1 macrophages have been designed to deliver IL-12, thereby increasing the M1/M2 ratio (359). For TAM reprogramming, proposed platforms include mannose-modified PEGylated PLGA nanoparticles, L-α-phosphatidylcholine/DSPE-PEG2000-amine formulations, and kinase-inhibiting amphiphiles that target NF-κB/MAPK or the CSF1-CSF1R axis. In addition, IRF5/IKKβ inhibitor of nuclear factor κ-B kinase subunit β (IKKβ) messenger RNA (mRNA)-loaded cationic poly (β-amino ester) nanoparticles reprogram M2 macrophages by promoting tumor-associated antigen release, activating MAPK signaling, and triggering M1 activation (360, 361).

Despite the promise, macrophage-targeted approaches face engineering challenges. Key issues include non-specific clearance or depletion of macrophages beyond TAMs, underscoring the need to spare TRMs (362, 363). Checkpoint modulation in TAMs can also provoke autoimmunity or therapeutic resistance. Thus, future nanomaterials should prioritize specificity for TAMs, controlled immune stimulation, and optimization to minimize off-target effects. Combining multiple mechanisms within a single nanoplatform may further enhance antitumor responses and limit progression and metastasis, while attention to non-invasive delivery and precise targeting remains essential.

Another promising angle is the microbiome. Gut bacteria influence macrophage numbers and the efficacy of PD-L1 immunotherapy. If we understand how microbes push macrophages toward M1 or M2 states, we could use them (delivered with nanoparticles) to reshape the TME and control TAMs (363–366). The microbiome is an important regulator of the tumor microenvironment, significantly influencing immune populations and immune regulation within the TME. The gut microbiome supports T-cell (including Treg) differentiation by releasing microbial metabolites and inflammatory mediators that contribute to immune suppression or activation. The microbiome and its metabolites also affect the efficacy of anticancer drugs. Both the gut and tumor-associated microbiomes can serve as biomarkers, and microbiome-informed strategies, including tumor-targeting bacteria and microbial-derived therapeutic agents, can be explored for tumor treatment. Conversely, the microbiome can contribute to cancer development and affect the response to therapy by releasing metabolites that can damage DNA. The microbiome can also regulate the TME by inducing inflammation that favors tumor progression (367–369). This bidirectional interplay between immune cells and the microbiome offers opportunities to design therapeutic strategies that modulate immune activation and enhance antitumor immunity within the TME, thereby controlling tumor growth.

In conclusion, macrophage-engineered therapies are highly promising for tumor targeting, but they require rigorous evaluation and rational combinations that act within the TME while minimizing effects on normal tissues. More broadly, monocyte-macrophage plasticity represents a central organizing principle of tumor immunology and an emerging therapeutic vulnerability for reshaping the tumor immune microenvironment.

## Figures and Tables

**Table 1.** Monocyte and macrophage (TAMs) receptors and their function.

Receptor	Function
PD-L1	Inhibits T-cell activation and promotes immune evasion
TLRs	Induce M1-like macrophage polarization and inflammatory cytokine production
CD40	Activates macrophages and dendritic cells to promote antitumor T-cell responses
CD137 (4-1BB)	Induces pro-inflammatory cytokine secretion and regulates IL-10 production
Mannose receptor (CD206)	Marker of immunosuppressive, M2-like TAMs
MARCO	Promotes pro-inflammatory signaling and antitumor macrophage reprogramming
Folate receptor-β (FOLR2)	Supports immunosuppression and limits CD8^+^ T-cell infiltration
EGFR	Promotes tumor cell proliferation, invasion, and metastasis

**Table 2.** Monocyte and macrophage (TAM) cytokines/chemokines and their functions.

Cytokine / Chemokine	Function
CCL1	Recruits TAMs and promotes tumor progression
CCL2	Recruits monocytes to the tumor microenvironment
CCL5	Promotes macrophage migration and tumor infiltration
CCL17 / CCL22	Recruit regulatory T cells (Tregs)
CCL18	Recruits naïve T lymphocytes and promotes immunosuppression
CXCL10	Recruits CXCR3^+^ effector T cells and supports Th1 antitumor responses
CXCL12	Directs TAM migration to hypoxic TME niches and promotes M2 polarization
IL-6	Activates NF-κB and STAT signaling to promote tumor progression and M2 polarization
IL-10	Induces M2 macrophage polarization and immunosuppression
IL-33 / IL-34	Promote M2 macrophage polarization
TGF-β	Suppresses early tumor growth but later expands Tregs and M2 macrophages
M-CSF (CSF1)	Promotes TAM recruitment, survival, and differentiation

## Data Availability

“No datasets were generated or analyzed in the current study.”
